# Estimates of genomic heritability and genome-wide association studies for blood parameters in Akkaraman sheep

**DOI:** 10.1038/s41598-022-22966-8

**Published:** 2022-11-02

**Authors:** Yunus Arzik, Mehmet Kizilaslan, Stephen N. White, Lindsay M. W. Piel, Mehmet Ulas Cinar

**Affiliations:** 1 Department of Animal Science, Faculty of Agriculture, Erciyes University, 38039 Kayseri, Türkiye; 2 International Center for Livestock Research and Training, Ministry of Agriculture and Forestry, 06852 Ankara, Türkiye; 3 Department of Veterinary Microbiology and Pathology, College of Veterinary Medicine, Washington State University, Pullman, WA 99164 USA; 4 USDA-ARS Animal Disease Res., 3003 ADBF, WSU Pullman, WA 99164 USA; 5 Present Address: Genus, DeForest, WI 53532 USA

**Keywords:** Animal breeding, Genetic association study, Genetic markers, Genomics, Immunogenetics

## Abstract

The aim of this study was to estimate genomic heritability and the impact that genetic backgrounds have on blood parameters in Akkaraman sheep by conducting genome-wide association studies and regional heritability mapping analysis. Genomic heritability estimates for blood parameters ranged from 0.00 to 0.55, indicating that measured phenotypes have a low to moderate heritability. A total of 7 genome- and 13 chromosome-wide significant SNPs were associated with phenotypic changes in 15 blood parameters tested. Accordingly, *SCN7A*, *SCN9A*, *MYADM-like*, *CCDC67*, *ITGA9*, *MGAT5*, *SLC19A1, AMPH*, *NTRK2*, *MSRA, SLC35F3*, *SIRT6*, *CREB3L3,* and *NAV3* genes as well as three undefined regions (LOC101117887, LOC106991526 and LOC105608461) were suggested as candidates. Most of the identified genes were involved in basic biological processes that are essential to immune system function and cellular growth; specific functions include cellular transport, histone deacetylation, cell differentiation, erythropoiesis, and endocytosis. The top significant SNP for HCT, MCH, and MCHC was found within a genomic region mainly populated by the *MYADM-like* gene family. This region was previously suggested to be under historical selection pressure in many sheep breeds from various parts of the world. These results have implications on animal breeding program studies due to the effect that the genetic background has on blood parameters, which underlying many productive and wellness related traits.

## Introduction

Sheep (*Ovis aries*) were one of the earliest species to be domesticated by humans^1^. This domestication can be seen spread across various geographical settings and expanded over many continents. Native sheep breeds are important genetic resources due to their adaptive capacities built up for centuries and today they have excellent gene-environment interaction in terms of survivability^2^. Among the domestic sheep breeds in Türkiye, the Akkaraman sheep is one of the most widespread fat-tailed breeds, owing to its features that make it well-suited for arid environments and utilization of low-quality pastures like various other fat-tailed sheep^3–8^.

Complete Blood Counts (CBCs) allow for the examination of blood parameters and are widely used to obtain information on the general health status of mammals^9^. Alterations in baseline CBC values provide vital information on the diagnosis of many diseases in farm animals and is one of the most frequently used diagnostic tools by veterinarians^10^. Furthermore, cells contained within blood perform vital tasks associated with animal survival and disease resistance^11^. Measured blood parameters assess three major cell groups consisting of erythrocytes (total red blood cell, total hemoglobin, percent hematocrit, mean corpuscular volume, mean corpuscular hemoglobin, mean corpuscular hemoglobin concentration, and red blood width ), leukocytes (total white blood cell, neutrophils, lymphocytes, monocytes, eosinophils and basophils) and platelets (total platelet count, mean platelet volume, and platelet width distribution)^12^.

Environmental factors such as regional pathogens, nutrition, and altitude are known to have a significant impact on blood parameters in sheep^13^. In addition to these, genetics also play an important role in the blood parameters of sheep^14^. Genomic regions contributing to the determination of quantitative phenotypic characters, such as blood parameters, are termed as quantitative trait loci (QTL)^14–16^. Although, studies on the genetic mechanisms responsible for alterations of blood parameters in sheep are quite scarce, the release of a sheep reference genome (Oar_v4.0) enabled researchers to identify genetic loci underlying changes in CBC values at the whole-genome level^14,15,17–19^. One such study completed a genome-wide association study for red blood cell phenotypes in three domestic sheep breeds, namely Columbia, Polypay, and Rambouillet, where a significant single nucleotide polymorphism (SNP) was found to be associated with mean corpuscular hemoglobin concentration. Further analysis determined that variants within the *MYADM*-*like* gene family were likely responsible for the red blood cell (RBC) abnormalities seen in sheep enrolled in the study^15^. A separate genome-wide association study (GWAS) assessed erythrocyte-related traits in Alpine Merino sheep, where two particular genes (i.e., *PLCB1* and *FLI1*) were found to be associated with hematopoiesis, or the production of cells contained within blood^14^. The aim of this study was to investigate the genetic basis of 18 phenotypes associated with erythrocytes, leukocytes, and platelets in Akkaraman sheep. For this purpose, genetic parameter estimates, a genome-wide association analysis and regional heritability mapping analysis were performed using phenotypes obtained from CBCs and genotypes obtained from a 50 K ovine SNP panel.

## Results

### Descriptive statistics

Outliers in the phenotypic observations for each trait were removed from the data. Table 1 contains the abbreviations used for each of the 18 phenotypic traits measured. More detailed information about the phenotype data is presented in Table 1.

**Table 1:** Descriptive statistics of blood parameters.

Parameters	Abr	N	Mean ± SE	Min	Max
Red blood cell (10^12^/l)	RBC	478	5.43 ± 0.12	0.31	10.70
Hemoglobin (g/dl)	HGB	468	9.51 ± 0.05	6.60	12.20
Hematocrit (%)	HCT	478	23.25 ± 0.45	4.20	44.20
Mean corpuscular volume (fl)	MCV	476	45.19 ± 0.24	35.70	58.40
Mean corpuscular hemoglobin (pg)	MCH	475	24.16 ± 6.90	1.60	71.50
Mean corpuscular hemoglobin concentration (g/dl)	MCHC	477	50.86 ± 1.22	3.60	139.70
RBC volume distribution width coefficient of variation	RDW_CV	476	14.30 ± 0.31	2.00	35.10
RBC volume distribution width standard deviation	RDW_SD	477	23.32 ± 0.50	0.00	57.00
White blood cell (10^9^/l)	WBC	477	9.68 ± 0.18	0.85	18.28
Neutrophils (%)	NEU	477	33.65 ± 0.85	4.80	85.60
Lymphocytes (%)	LYM	474	61.32 ± 0.72	21.10	92.30
Monocytes (%)	MON	382	0.94 ± 0.03	0.00	4.60
Eosinophils (%)	EOS	457	0.63 ± 0.02	0.00	2.20
Basophils (%)	BAS	466	0.42 ± 0.01	0.00	1.20
Platelets (10^9^/l)	PLT	214	455.19 ± 8.96	105.00	819.00
Mean platelets volume (fl)	MPV	425	12.24 ± 0.29	4.70	21.10
Procalcitonin (ng/ml)	PCT	209	0.29 ± 0.006	0.07	0.51
Platelets distribution width (fl)	PDW	419	15.37 ± 0.05	12.50	18.60

N is the number of observations; SE is standard error.

### Genomic heritability estimates

Heritability estimates for blood parameters ranged from 0.00 to 0.55, indicating a low to moderate heritability for measured phenotypes in Akkaraman sheep. Erythrocyte traits had heritability estimates ranging from 0.07 to 0.23, while leukocyte related traits tended to have a broader range with 0.02 for EOS (%) and 0.55 for WBC (10^9^/l). Lastly, heritability estimates for platelet related traits ranged between 0.00 and 0.13, where the former was found for PCT (ng/ml) and the later for PLT (10^9^/l). The variance components and genomic heritability estimates with their standard errors are presented in Table 2.

**Table 2:** Variance components and genomic heritability (h^2^) estimates for the traits.

Parameters	Vg	Ve	Vp	h^2^ ± SE
RBC	1.48	5.93	7.41	0.20 ± 0.10
HGB	0.20	1.38	1.58	0.12 ± 0.08
HCT	21.46	76.38	97.84	0.21 ± 0.10
MCV	6.80	41.60	48.40	0.14 ± 0.09
MCH	70.04	270.34	340.38	0.20 ± 0.10
MCHC	173.39	569.22	742.61	0.23 ± 0.10
RDW_CV	8.47	76.00	84.47	0.10 ± 0.08
RDW_SD	13.90	164.91	178.81	0.07 ± 0.07
WBC	8.58	6.98	15.56	0.55 ± 0.13
NEU	54.26	290.61	344.87	0.15 ± 0.09
LYM	38.13	227.67	265.80	0.14 ± 0.09
NEU/LYM	0.08	0.31	0.39	0.20 ± 0.10
MON	10.83	37.18	48.01	0.22 ± 0.10
EOS	0.03	1.40	1.43	0.02 ± 0.06
BAS	0.05	0.13	0.18	0.27 ± 0.01
PLT	2265.67	14,853.48	17,119.15	0.13 ± 0.10
MPV	4.09	32.07	36.16	0.11 ± 0.09
PCT	0.00	354.66	354.66	0.00 ± 0.12
PDW	0.07	1.58	1.65	0.04 ± 0.07

Vg is additive genetic variance; Ve is residual variance; Vp is total phenotypic variance; h^2^ is genomic heritability; SE is standard error.

### Genome-wide association analyses

A linear mixed model was used to estimate the effects of fixed factors before proceeding with the GWAS analysis. Accordingly, sex, herd, and age (in days) were observed to have significant effects on WBC and RDW_CV traits. Concurrently, the live weight of animals was found to be statistically significant for NEU, LYM, EOS, and MCH. Finally, the interactions between herd and feeding type as well as, sex and feeding type, were found to be important for WBC and LYM respectively. Therefore, these were included in the GWAS models.

**Figure 1:**
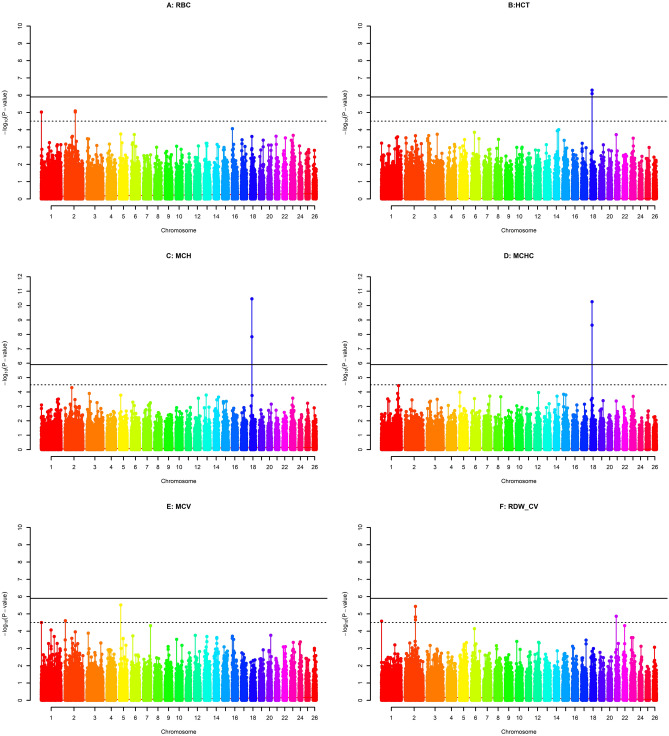
Manhattan plots of GWAS for six erythrocyte traits. Red dashed lines (i.e., upper line) represent Bonferroni-corrected genome-wide significance level, while black dashed lines (i.e., lower line) are for Bonferroni-corrected chromosome-wide significance level. The six traits were: red blood cell count (**A**), hematocrit (**B**), mean corpuscular hemoglobin (**C**), mean corpuscular hemoglobin concentration (**D**), mean corpuscular volume (**E**) and RBC volume distribution width coefficient of variation (**F**).

**Figure 2:**
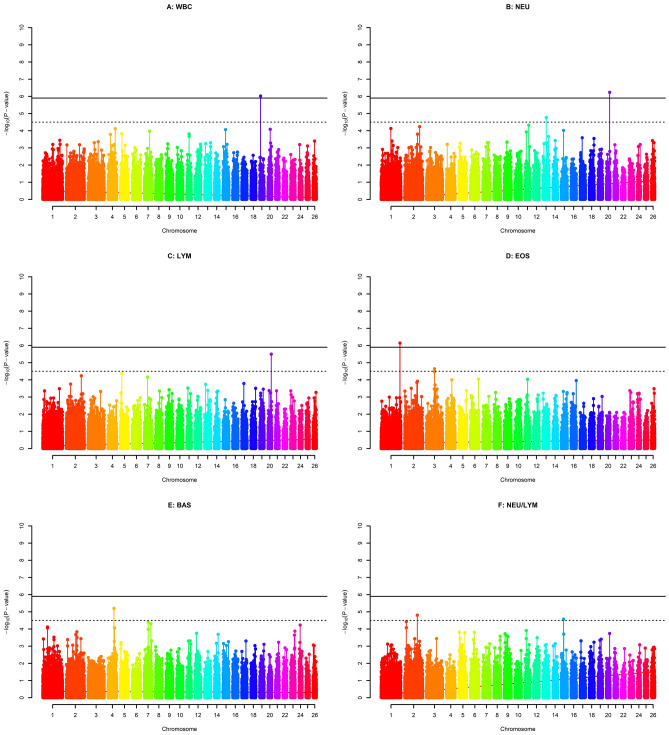
Manhattan plots of GWAS for leukocyte related traits. Red dashed lines (i.e., upper line) represent Bonferroni-corrected genome-wide significance level, while black dashed lines (i.e., lower line) are for Bonferroni-corrected chromosome-wide significance level. White blood cell count (**A**), neutrophils (**B**), lymphocytes (**C**), eosinophils (**D**), basophils (**E**) and neutrophils/lymphocytes (**F**).

**Figure 3:**
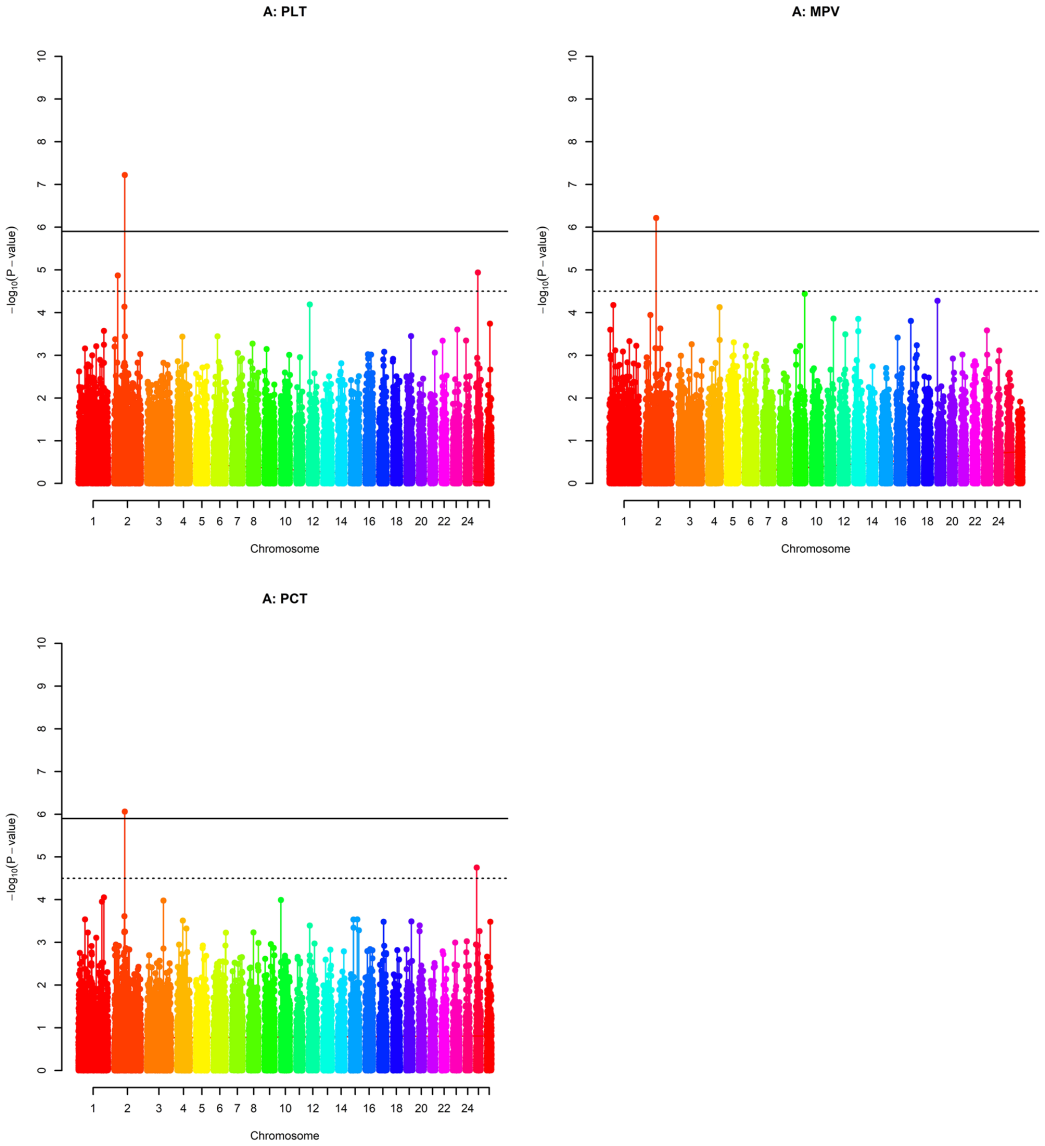
Manhattan plots of GWAS for thrombocyte, platelet, traits. A red dashed line represents the Bonferroni genome-wide significance level and a black dashed line represents the chromosome-wide significance level. Platelets count (**A**), mean platelet volume (**B**), procalcitonin (**C**).

The quantile–quantile (Q-Q) plots of the observed test statistics for each SNP after and before genomic control were compared with the expected test statistics under the null hypothesis of no association (respectively see Supplementary Fig. 1 and 3). A Q-Q plot and the estimated inflation factor lambda (λ) were obtained for each phenotype which was set to 1 with genomic control applied. The corrected p-values for each trait were obtained as a result of the GWAS and were presented on –log10 scale in the Manhattan plots given in Figs. 1, 2, and 3. Within these figures, the genome- and chromosome-wide thresholds are presented by either red and black dashed lines respectively. Accordingly, a total of 7 genome-wide significant SNPs and 13 chromosome-wide significant SNPs were found by the GWAS conducted for 15 blood parameters of Akkaraman lambs. On the other hand, no significant or suggestive SNPs were found for 4 of the traits studied, namely HGB, RDW_SD, MON and PDV. More details on the significant SNPs were presented in Table 3.

**Table 3:** Significant SNPs associated with the phenotypes.

Traits	SNP name	Chr	Position (bp)	p-value	Vg (%)	MAF	Other associated phenotypes	Associated genes	
								Name	Distance (bp)
RBC	rs418188401	2	142,026,337	4.08 × 10^–06^	0.42	0.467	RDW_CV	*SCN7A*	Within
RBC	rs415321135	2	142,135,301	1.30 × 10^–05^	0.48	0.351	–	*SCN9A*	Within
HCT	rs402657016	18	19,382,508	9.59 × 10^–08^	1.12	0.166	MCH, MCHC	*–*	–
HCT	rs424122039	18	19,330,647	1.61 × 10^–07^	1.06	0.170	MCH, MCHC	*MYADM-like*	Within
MCH	rs424122039	18	19,330,647	3.00 × 10^–08^	2.96	0.170	HCT, MCHC	*MYADM-like*	Within
MCH	rs402657016	18	19,382,508	5.84 × 10^–07^	2.20	0.166	HCT, MCHC	–	–
MCHC	rs424122039	18	19,330,647	4.83 × 10^–11^	2.15	0.170	HCT, MCH	*MYADM-like*	Within
MCHC	rs402657016	18	19,382,508	2.07 × 10^–09^	1.82	0.166	HCT, MCH	*–*	–
MCV	rs427024436	5	17,364,739	3.03 × 10^–06^	0.57	0.265	–	*SIRT6, CREB3L3*	Near
RDW_CV	rs415321135	2	142,135,301	3.66 × 10^–06^	0.67	0.398		*SCN9A*	Within
RDW_CV	rs424460878	21	10,141,822	1.36 × 10^–05^	0.67	0.366	–	*CCDC67*	Within
RDW_CV	rs409151807	2	142,204,729	1.48 × 10^–05^	0.68	0.349	–	*SCN9A*	Within
RDW_CV	rs418188401	2	142,026,337	2.20 × 10^–05^	0.40	0.467	RBC, HCT	*SCN7A*	Within
WBC	rs423900300	19	11,192,604	1.19 × 10^–06^	0.74	0.302	–	*ITGA9*	Within
NEU	rs419009933	20	45,260,345	8.94 × 10^–07^	4.35	0.097	LYM	*LOC101117887*	Within
NEU	rs422235744	13	43,611,954	1.75 × 10^–05^	1.57	0.275	–	*LOC106991526*	Within
LYM	rs419009933	20	45,260,345	3.19 × 10^–06^	3.07	0.097	NEU	*LOC101117887*	Within
NEU/LYM	rs416257223	2	175,533,494	1.81 × 10^–05^	1.51	0.091		*MGAT5*	Within
EOS	rs429955018	1	263,341,417	3.05 × 10^–07^	18.32	0.056	–	*SLC19A1*	Within
EOS	rs424356478	3	112,927,310	3.32 × 10^–05^	1.98	0.466	–	*NAV3*	Near
BAS	rs417370910	4	82,433,190	6.26 × 10^–06^	1.00	0.341	–	*AMPH*	Within
PLT	rs419857573	2	99,213,936	1.76 × 10^–07^	2.73	0.296	PCT	*LOC105608461*	Within
PLT	rs410481204	2	34,556,977	1.20 × 10^–05^	1.83	0.370	–	*NTRK2*	Within
MPV	rs424459233	2	102,985,180	1.51 × 10^–06^	5.24	0.199	–	*MSRA*	Within
PCT	rs419857573	2	99,213,936	8.10 × 10^–07^	7.58	0.296	PLT	*LOC105608461*	Within
PCT	rs426696259	25	6,748,890	8.73 × 10^–06^	4.40	0.407	–	*SLC35F3*	Within

SNP position based on OAR_v4.0 assembly; Vg (%) is additive genetic variance explained by each SNP; MAF is minor allele frequency.

### Regional heritability mapping

RHM analysis results using 5 Mb windows are presented in detail in Supplementary Table 1. A total of 30 regions, 22 of which were genome-wide significant, were found to be associated with blood parameters. The genome-wide significant regions on OAR1 (between 270,665,267 and 273,024,024) and OAR18 (between 17,367,201 and 19,881,168) were found to be common for MCH and MCHC traits. In addition, the region between positions 50,907,002 and 53,876,295 on OAR6 was found to be significant for NEU, LYM and NEU/LYM traits. Regional heritabilites for windows were ranged from 0.05 to 0.26. While LRT values for the traits were varied between 13.99 and 49.15, p-values of the significant windows at -log10 scale were ranged from and from 4.03 to 11.92, respectively.

### Candidate genes and QTLs

Regarding the significant SNPs, the nearest genes and/or the genes flanked by two consecutive significant SNPs (e.g., *rs428421565* and *rs424356478)* were found on Oar_v4.0 via NCBI genome data viewer. As a result, 11 genome-wide and chromosome-wide significant SNPs were observed to be within specific genes, namely Sodium Voltage-Gated Channel Alpha Subunit 7 (*SCN7A*), Sodium Voltage-Gated Channel Alpha Subunit 9 (*SCN9A*), Myeloid-associated Differentiation Marker-like (*MYADM-*like), Coiled-Coil Domain-Containing Protein 67 (*CCDC67*), Integrin Subunit Alpha 9 (*ITGA9*), Alpha-1,6-Mannosylglycoprotein 6-Beta-N-Acetylglucosaminyltransferase (*MGAT5*), Solute Carrier Family 19 Member 1 (*SLC19A1*), Amphiphysin (*AMPH*), Neurotrophic Receptor Tyrosine Kinase 2 (*NTRK2*), Methionine Sulfoxide Reductase A (*MSRA*) and Solute Carrier Family 35 Member F3 (*SLC35F3*) on OAR chromosomes 1, 2, 4, 18, 19, 21 and 25. In contrast, 3 of the significant SNPs were found in close proximity to Sirtuin 6 (*SIRT6*), CAMP Responsive Element Binding Protein 3 Like 3 (*CREB3L3*) and Neuron Navigator 3 (*NAV3*) on OAR chromosome 3 and 5. Also 3 of the significant SNPs were within unidentified region namely LOC101117887, LOC106991526 and LOC105608461) on OAR chromosome 2, 13 and 20.

## Discussion

The acceleration of technological advancements in the field of genomics, followed by the assembly of reference genome sequences of different species, has been greatest the contribution to the identification of genes associated with complex traits^20–22^. Furthermore, the genetic background of economically important livestock traits such as disease resistance, milk yield, meat yield, and hematological parameters have been revealed by associating phenotype and genotype data with a linear mixed model based analysis, which is the commonly used approach in GWAS^7,15,17,23,24^. In this study, genomic heritability estimates for 19 blood parameters were obtained in Akkaraman sheep, which is a widespread indigenous sheep breed in Türkiye. Genome-wide association analyses for each trait was implemented, which led to the suggestion of 19 specific genes and 3 unidentified locations (Table 3).

There are highly variable results from the few studies conducted on blood parameters in sheep^14,25–27^. For instance, a study conducted on Santa Inês sheep found the mean values for RBC, PLT, WBC, HCT, and HGB traits to be 9.97, 383.8, 10.96, 29.05, and 8.43 respectively^26^. In the current study, these values ​​were found to be similar for HGB (9.51) and WBC (9.68); however, for PLT (455.0) and HCT (23.25), the values ​​were found to be higher. Finally, the mean value for RBC (5.43) was found to be lower. In another study conducted on Alpine Merino sheep, the means for MCH, MCHC, and RWD_CV were found to be 13.24, 37.74, and 0.39 which are all substantially lower than the means found in the present study [MCH (24.16), MCHC (50.86) and RWD_CV (14.30)]. The possible differences for the results can be attributed to different environmental (i.e., climate, ecotype, altitude), physiological, age, and health-related conditions as well as different genetic backgrounds of the breeds.

In the present study, low to moderate (from 0.00 to 0.55) heritability estimates were found for blood parameters (Table 2). Studies conducted on the estimation of heritabilities for blood parameters of sheep are quite scarce. In a study of Santa Inês sheep, heritability estimates using the genomic relationship matrix were found to be 0.18, 0.17, 0.22, 0.20, and 0.16 for the RBC, PLT, WBC, HCT, and HGB traits respectively^28^. Results in the current study were similar for RBC (0.20), PLT (0.13), HCT (0.21), and HGB (0.12), but higher for WBC (0.55). Again, for Santa Inês sheep, the heritability estimation for HCT (0.18) was found to be compatible with our current study^29^. Additive genetic variance of the traits and, accordingly, heritability is affected by many natural factors such as natural selection, mutation, genetic drift, and genetic migration as well as artificial selection^30^. Populations exposed to these natural and artificial factors for different periods of time and in various manners can be expected to have differing heritability for the same trait. However, the heritability estimates of many traits in our study were quite similar with the results of previous studies, albeit these studies contained different breeds. On the other hand, comparatively small sample size of our study led to the relatively high standard errors for the heritability estimations which might rather hamper finding differences between estimations from different studies. However, in any case, the concordant moderate heritability estimates of many blood parameters in different sheep breeds might lead one to speculate that there has not been any systematic selection applied to these parameters which might alter the share of genetic variance in the phenotypic characteristics of these animals. Thus, the genetic improvement of these parameters by selection strategies is quite possible.

As a result of the GWAS conducted in this study, a total of 20 SNPs were found to be significant for 15 different blood parameters, with 7 being genome-wide and 13 being chromosome-wide. No significant SNPs were found for HGB, RDW_SD, MON and PDW traits. The current study agrees with those prior, which show that the number of SNPs in peaks obtained for complex traits in sheep is low during GWAS analyses^14,15,17,24,31^. The main reasons for this are suggested to be the relatively short linkage disequilibrium in the sheep genome and the highly polygenic structure of measured traits^32,33^.

Erythrocytes are an important cell type as they directly affect performance and growth as well as the health status of animals^34^. The present GWAS determined that 6 erythrocyte-related traits were significantly associated with 8 SNPs, which were made up of 2 genome-wide and 6 chromosome-wide SNPs on OAR chromosome 1, 2, 5, 18 and 21 (Table 3). Importantly, among those, SNPs with pleotropic effects were observed (*rs424122039*, *rs402657016,* and *rs418188401*) for RBC, HCT, MCH, MCHC, and RDW_CW traits. The top associated SNP regarding all measured CBC traits was observed for MCHC (*p* = 4.83e-11), which was *rs424122039* on OAR chromosome 18 (Fig. 1 and Table 3). Furthermore, two other significant SNPs for MCH and HCT (*rs424122039*, *rs402657016*) were found to be adjacent to that defined for MCHC. Importantly, these SNPs were within an approximately ~ 52 Kb distance and discovered in the *MYADM-*like gene family on OAR chromosome 18. Interestingly, the association between the *MYADM*-like gene family and variable RBC parameters in sheep has been previously reported by Gonzalez et al.^15^. In the multi-breed GWAS, the discovered region and *rs424122039* SNP were specifically found to be associated with MCHC of three sheep breeds namely Columbia, Polypay, and Rambouillet. The region carrying the *MYADM*-like gene family has been reported to be conserved throughout many mammalian species (i.e., cow, pig, goat)^35,36^. Protein products synthesized by the *MYADM* gene family have been reported to be involved in membrane formation and regulatory processes in myeloid cell lines^37,38^. In addition to these functions, the increase of *MYADM* gene expression levels in pluripotent cells is involved in the completion of erythropoiesis^38^. The genomic region containing the significant SNPs encompasses a nearly 800 Kb window and was found to be a gene-rich region mainly populated by Myeloid-associated Differentiation Marker-like (*MYADM-like*) protein coding genes, pseudogenes, and other genes with a high number of SNP variants (Supplementary Fig. 2). This same region was also found to be significant in selection signature analyses during the ovine HapMap project, which was carried out with 74 sheep breeds from many parts of the world^32^ and by Abied et. al. with thin- and fat-tailed sheep breeds from Sudan and China^39^. The extreme historical selection pressure of the genomic region emphasizes that this region provides superiority in the adaptive and survival capacity of sheep. Although, the top SNP and the region of interest has been previously associated with the MCHC parameter^15^, this is the first study discovering the association between *rs424122039* and other red blood cell traits (i.e., MCH and HCT) in sheep.

Other SNPs associated with the erythrocyte-related traits were within genes Sodium Voltage-Gated Channel Alpha Subunit 7 (*SCN7A*) and Sodium Voltage-Gated Channel Alpha Subunit 9 (*SCN9A*), which were found to have pleiotropic effects associated with RBC and RDW_CV traits. This SCN gene family encodes for voltage-gated sodium channel proteins which are mainly involved in ion transport (GO:0,006,811), especially sodium ion transport (GO:0,006,814), in mammalian cells.

Three genome-wide and four chromosome-wide significant SNPs were associated with leukocyte related traits on OAR chromosome 1, 2, 3, 4, 13, 19 and 20 (Fig. 2 and Table 3). The SNP *rs423900300* was found to be associated with total WBC count and is located within the Integrin Subunit Alpha 9 (*ITGA9*) gene on OAR chromosome 19. This gene encodes an alpha integrin protein which mediates cell–cell and cell–matrix adhesion. Involved biological processes include cell adhesion (GO:0,007,155), integrin-mediated signaling pathway (GO:0,007,229), extracellular matrix organization (GO:0,030,198), and neutrophil chemotaxis (GO:0,030,593). In a study of breast cancer in humans, the expression of *ITGA9* was downregulated and lost in the majority of breast cancer cell line^40^. On the other hand, SNP *rs419009933* within *LOC101117887* on chromosome 20 showed pleotropic effect on NEU and LYM traits. Interestingly, this SNP had a negative effect on NEU with 4.35% of additive genetic variance, whereas it had positive effect on LYM accounting for3.07% of additive genetic variance (Table 3). Additionally, the Alpha-1,6-Mannosylglycoprotein 6-Beta-N-Acetylglucosaminyltransferase (*MGAT5*) gene was found to be related to the neutrophil to lymphocyte ratio (NEU/LYM). *MGAT5* encodes a protein belonging to the glycosyltransferase family and acts in biological processes such as the positive regulation of cell migration (GO:0,030,335) and the positive regulation of the STAT signaling pathway (GO:1,904,894). Previously, Bahaie et al. determined that deficiency of *MGAT5* cause increased neutrophilic inflammation in allergen challenged mice^41^. This gene also was found to be associated with T-cell glycosylation and plasma composition of the IgG glycome in ulcerative colitis in humans^42^.

SNP *rs429955018* on OAR chromosome 1 was associated with EOS with relatively high additive genetic variance (18%). This SNP was located within the Solute Carrier Family 19 Member 1 (*SLC19A1*) gene. This gene is involved in many biological processes such as folic acid transport (GO:0,015,884) and vitamin metabolic processes (GO:0,006,766). It is well known that folic acid and other vitamins have a critical role in the production of white blood cells, particularly granulocytes^43^. Finally, SNP *rs417370910,* present within the Amphiphysin (*AMPH*) gene on OAR chromosome 4, was associated with BAS (Fig. 2). *AMPH* is mainly involved in endocytosis (GO:0,006,897). Amphiphysin is one of the adhesion proteins, and used in many cells that perform endocytosis, which is a process regarding immune system cells (i.e., basophiles, macrophages) that is important in destroying the antigens (i.e., soluble antigens)^44^.

Within the platelet traits studied, there were two genome-wide and three chromosome-wide significant SNPs detected for PLT, MPV and PCT on chromosome 2 and 25 (Fig. 3 and Table 3). SNP *rs419857573* was within undefined region *LOC105608461 *and had pleotropic effects on PLT and PCT, explaining 2.73% and 7.58% of the additive genetic variance, respectively (Table 3). Besides that, *rs424459233* within the Methionine Sulfoxide Reductase A (*MSRA*) gene on OAR chromosome 2 was associated with MPV with 5.24% of additive genetic variance.

Regional heritability mapping approach was used in addition to the single step GWAS method to determine the existence of the association between specific genomic regions obtained from the genome and blood parameters. The RHM approach is widely used method to detect potentially important regions in the genome. It has been reported that it is a more effective method than the SNP-based GWAS method in detecting genomic regions containing multiple QTLs and rare allele with relatively moderate and small effects or epistatic effects in the genome^45–47^. In our study, as expected, RHM results validated the result of single step GWAS by sharing significant SNP located in the significant regions. However, some of the significant regions were not include significant SNP detected in GWAS (Supplementary Table 1 and Supplementary Fig. 4). The regions on OAR16 and 23 were found to be highly significant for MCV and RDW_CV traits, respectively (Supplementary Fig. 4). Interestingly, no SNP or QTL were found around these two regions for these two corresponding traits. Particularly, the region (between 38,337,897 and 40,409,152 positions) on OAR16 was associated with the MCV trait with having 45.36 LRT value and 11.08 -log10 scale p-value. Therefore, RDW_CV with the region on OAR23 (between 52,374,331 and 53,898,104 position) was found to be significant with the highest LRT and -log10 scale p-value as 49,15 and 11.92, respectively. Unlike single SNP GWAS analyzes, the region (between 50,907,002 and 53,876,295 position) on OAR6 was associated with NEU, LYM and NEU/LYM traits of white blood cells. It was demonstrated that when related SNPs do not have significant effects at the genome-wide level, RHM outperformed a conventional GWA study. In the RHM approach of the estimating heritability for a specific region, the estimate of additive variance is contributed by rare variants in addition to common variants^48^. Since RHM uses many sources of variance in regional heritability estimation, it allows the detection of loci that could not be found in single step GWAS analyses. In this context, the regions found in OAR16 and 23, which are important for MCV, RDW_CV, traits, have a very high potential to contain the important rare variants.

The results of the present GWAS revealed many genes (i.e., *SCN7A, SCN9A, MYADM-*like*, ITGA9, MGAT5, SLC19A1, SLC35F3* and *AMPH*) which are important to basic biological functions such as cellular transport, histone deacetylation, cell differentiation, erythropoiesis, and endocytosis^37,38,40–42,44^. Some of the identified SNPs (i.e., *rs418188401*, *rs402657016*, *rs424122039*, *rs419009933* and *rs419857573*) were responsible for pleotropic effects on WBC, RDW_CV, HCT, MCH, MCHC, NEU, LYM, PLT, and PCT measured traits. Furthermore, SNP *rs424122039* was found to be associated with MCH, MCHC, and HCT, supporting prior work which linked MCHC measurements to the same gene-rich *MYADM*-*like* region^15^. Previous studies have also shown that this region is under historical selection pressure^32,39^. In this context, the *MYADM*-*like* gene was suggested by Gonzalez et. al., (2013) as a potential candidate gene associated with erythrocyte related traits (i.e., MCHC); moreover, the results of our current study confirmed this hypothesis. Our current study is one of the limited number of studies aimed at understanding the genetic components underlying blood parameters in sheep. The current study and future studies of its kind will shed light on the genetic region that effect measured blood traits and how to use these associations to further selection programs.

## Conclusion

As a conclusion, in our study, it has been illustrated that 7 genome-wide and 13 chromosome-wide significance SNPs in GWAS and 30 significant regions in RHM are associated with blood parameters in Akkaraman lambs, which is an important breed in Türkiye. These SNPs were associated with 14 genes namely *SCN7A, SCN9A, MYADM-*like*, SIRT6, REB3L3, CCDC67, ITGA9, MGAT5, SLC19A1, NAV3, AMPH, NTRK2, MSRA, SLC35F3,* and three unidentified locations – (LOC101117887, LOC105608461 and LOC106991526). The results of this study can be utilized as a selection criterion in sheep breeding approaches. Considering that erythrocytes and leukocytes are directly related to adaptation and survivability of sheep, the resultant breeding approaches may produce more resistant and sustainable animals. Finally, in order to gain more sophisticated knowledge on the genetic mechanisms of blood parameters and transfer this knowledge to applications, it is strongly recommended that this and similar studies be conducted on larger numbers and in different populations.

## Material and methods

### Animal materials and phenotypes

The study was carried out in the outskirts of Ankara province, Türkiye (39°41' N; 33°01' E). The region is known to have poor pasture with a continental climate that has cold, snowy, winters and hot, dry, summers. Annual rainfall, average temperature, and average altitude are 389 mm, 11.7 °C, and 938 m respectively. In the study, 480 Akkaraman lambs (186 males and 294 females), were randomly selected from 3 different farms that are registered to the National Community-based Small Ruminant Breeding Program. Lambs were born between January and February of 2021 and weaned at an average age of 3 months old. After weaning, 372 lambs were grazed on the pasture during the summer period without supplemental feeding, while 114 lambs were subjected to a 90 days of finishing period with 750–1000 g concentrate feed/day. Selection decisions of the studied populations were given around the mating period (e.g., August–September) based on the phenotypically observed growth rates without any pedigree based selection.

Lambs were monitored from birth until the sample collection, which was around 6 months of age. Additional records including environmental factors (sex, herd, birth type, feeding type, pasture) were collected. Animals with a clear sign of an active disease were excluded from the study. 6 ml of blood were taken from the *V. jugularis* in an EDTA-coated vacutainer and transferred to the laboratory in cooling box. Samples were analysed within 6 h of collection to avoid hemolysis of blood. The 18 blood parameters were measured by ZETA Laboratories (Ankara, Turkiye). Eight of these phenotypes were erythrocyte phenotypes including total red blood cell count (RBC, 10^12^/dl), hemoglobin (g/dl), hematocrit (%), mean corpuscular volume (fl), mean corpuscular hemoglobin (pg), mean corpuscular hemoglobin concentration (g/dl), coefficient of variation of RBC volume distribution width, and standard deviation of RBC volume distribution width. Leukocyte phenotypes were total white blood cell count (10^9^/dl), neutrophils (%), lymphocytes (%), monocytes (%), eosinophils (%), and basophils (%). Lastly, platelet (PLT) phenotypes were total platelet count (10^9^/dl), mean platelet volume (fl), procalcitonin (ng/ml) and platelet distribution width (fl). Preceding further analyses, outliers were identified and observations exceeding three standard deviations ± mean for each trait were excluded from the data. Descriptive statistics of these parameters were presented in Table 1.

### DNA extraction and genotyping

After the analyses for blood parameters, the remaining samples were transferred to the Genetics Laboratory of the International Center for Livestock Research and Training (ICLRT) for extraction of DNA. To minimize contamination, DNA extraction was performed with the Qiacube HT automated device using a commercial kit (Qiagen Blood kit, Hilden, Germany). Quality control of the extracted DNA was performed before genotyping. Samples that passed the quality criteria (A260/280 > 1.8, A260/230 > 1.5, > 70 ng/µl, high DNA integrity) were stored for genotyping at -20 °C. Genotyping was performed using the Axiom 50 K Ovine Genotyping Array with the GeneTitan MultiChannel microarray instrument (Thermo Fisher Sci, Waltham, MA, USA) at the Genetics Laboratory of the ICLRT. The genotyping procedure was implemented according to the manufacturer’s protocol.

### Quality control (QC)

After the genotyping process, filtering was performed with certain QC criteria to decrease Type-I and Type-II error rates. QC criteria adopted by McCarthy et al., (2008), The Wellcome Trust Case Control Consortium, (2007), and Weale, (2010) were considered^49–51^. Accordingly, SNPs with minor allele frequency (MAF) lower than 0.05, a call rate below 95%, and SNPs that are mapped on sex chromosomes were excluded from the data. Additionally, samples with call rate lower than 90% and samples with an Identity By State (IBS) value of more than 95% were removed from the data. Finally, SNPs deviating from Hardy–Weinberg Equilibrium (HWE) (i.e., p-value = 0.05/number of SNPs) were excluded from the data. The threshold value for the deviation from HWE was determined with the Bonferroni correction. Raw genotype data contained 49,931 SNPs and after performing QC analyses with the described criteria, 40,463 SNPs and 479 animals remained for further analyses.

### Statistical analyses

Since GWAS does not accommodate missing genotypes, an expected genotype score estimated from the population was used to impute missing genotypes, considering Chen and Abecasis (2007)^52^. In addition, the genomic relationship matrix (G), described by Astle and Balding (2009) and equivalent of model 2 provided by VanRaden (2008), was calculated to be used in the genetic parameter estimations and GWAS^53,54^.

Estimation of the variance components and narrow sense heritability (h^2^) for the traits were implemented using the general generalized linear model below and with restricted maximum likelihood (REML) approach using ‘sommer’ package of R^55^.

GWAS were performed with Mixed Linear Models to detect significant SNPs associated with phenotypes. In the model, the additive genetic effect of SNPs was estimated by including a genomic relation matrix in the model to also account for covariance between related individuals and population stratification. The following linear model (1) was used in the univariate analysis implemented with the R ‘GenABEL’ package^56^:

1 $${\mathbf{y}} = {{\varvec{\upmu}}} + {\mathbf{X }}\boldsymbol{\beta} + {\mathbf{Zu}} + {\mathbf{e}}$$

where **y** is the vector of individual observations of each blood parameter focused, **µ** is the population mean regarding the trait of interest, **β** is the vector of SNP and fixed environmental effects (i.e., sex, herd, and age (in days) for WBC and RDW_CV traits; the live weight of animals for NEU, LYM, EOS, and MCH; the interactions between herd and feeding type and the interaction between sex and feeding type for WBC and LYM), **u** is the polygenic background effect obtained from MVN (u ~ 0, **G**σ_u_^2^), and **e** is the vector of random residual errors obtained from MVN (e ~ 0, **I**σ_e_^2^). **X** and **Z** are the design matrices mapping fixed effects and polygenic background effects to each observation respectively.

The quantile–quantile plots were used to compare the observed test statistics for each SNP to those expected under the null hypothesis of “no association” to detect any inflation in the test statistics due to systematic biases. To avoid higher inflation in the test statistics, genomic control was applied to the p-values as described by Devlin and Roeder^57^. Manhattan plots were used to visualize the –log10 (p-value) of all SNPs respective to their position at the associated chromosome. Genome-wide and chromosome-wide significance thresholds defined by using Bonferroni correction were applied to avoid increasing Type 1 Error rate due to multiple testing SNPs. Accordingly, the genome-wide significance threshold was set to 1.23e-06 (0.05/40.463) and the chromosome-wide significance threshold was set to 3.21e-05 ((0.05/40.463) *26). Genetic variances regarding the significant SNPs were estimated with the formula below:

$$\user2{\% V}_{{\varvec{g}}} \left( {{\varvec{SNP}}} \right) = \frac{{2{\varvec{pqa}}^{2} }}{{{{\varvec{\upsigma}}}_{{\varvec{u}}}^{2} }} \times \textbf{100}$$

where ‘**p**’ is the major allele frequency, ‘**q**’ is the minor allele frequency and ‘${\varvec{a}}$’ is the estimated effect size of the corresponding SNP by the GenABEL R package.

In addition to SNP based GWAS analyzes, regional heritability mapping (RHM) analyzes of traits were performed to evaluate the effect of each predefined region^46,48^. For this purpose, each chromosome for RHM was divided into 5 Mb windows with 2.5 Mb overlap. After sliding windows, 1038 windows were obtained with average 40 SNPs. The whole genome relationship matrices and regional genomic relationship matrix were constructed with the SNP information by using Genome-wide Complex Trait Analysis (GCTA) software (version 1.94.1)^58^. Model 2 was used to detect the contribution of each window effect as follow up below:

2 $${\mathbf{y}} = {{\varvec{\upmu}}} + {\mathbf{X}\boldsymbol{\beta} } + {\mathbf{Zu}} + {\mathbf{Qr}} + {\mathbf{e}}$$

where **y, µ, β** and **u** were the same as Model 1. **r** is the background effect of each window obtained from MVN (r ~ 0, **Q**σ_r_^2^), and **Q** are the design matrices mapping fixed effects and polygenic background effect of the windows to each observation respectively. A likelihood ratio test (LRT) was used to compare full (2) and reduced (1) model to detect the impact of each window in GCTA software. Bonferroni correction test was applied to set genome-wide (p ≤ 0.05) and suggestive (p ≤ 0.10) significant levels. Regional heritability (h^2^_reg_) was calculated as $h_{reg}^{2} = \frac{{{\upsigma }_{reg}^{2} }}{{{\upsigma }_{p}^{2} }}.$

### Functional annotation

Positional information of significant SNPs and the nearby genes were obtained from NCBI Genome Data Viewer by using the Oar_v4.0 genome assembly^59^. Genes that are containing the SNPs were suggested as candidates. If the significant SNP was not within a gene, the nearest genes and the genes flanked by two consecutive significant SNPs were suggested as candidates. Biological information on the identified candidate genes were obtained by using DAVID Bioinformatics Resources 2021^60,61^. Additionally, previously identified QTL related to sheep blood parameters were checked through Animal QTL Database to see if there is any overlap with the SNPs identified in this study^62^. Where there is not enough annotation in the sheep genome reference, orthology between species was utilized and annotation of the specific genes from cattle, goats, mice, and humans were used. Finally, the biological processes involved by the genes of significant SNPs were represented with their Gene Ontology (GO) terms, which can be further detailed with QuickGo website^63^.

### Ethics statement

All experimental protocols in the study was approved and conducted under the supervision of Animal Experiments Local Ethics Committee of the International Centre for Livestock Research and Training (Approval Number: 20.11.2020–183), Ankara, Türkiye, and the methods were also carried out in accordance with the ARRIVE guidelines.

## Supplementary Information

Supplementary Information 1.

Supplementary Information 2.

Supplementary Information 3.

Supplementary Information 4.

Supplementary Information 5.

## Data Availability

The data generated during the current study is available from the corresponding author on reasonable request.
