# Supplementary material for: Estimates of genomic heritability and genome-wide association studies for blood parameters in Akkaraman sheep
Source: Sci Rep. 2022 Nov 2;12:18477. doi: 10.1038/s41598-022-22966-8 (PMC9630504; doi:10.1038/s41598-022-22966-8)
Supplement: Supplementary file 1 — Supplementary Information 1. [file 41598_2022_22966_MOESM1_ESM.pdf]

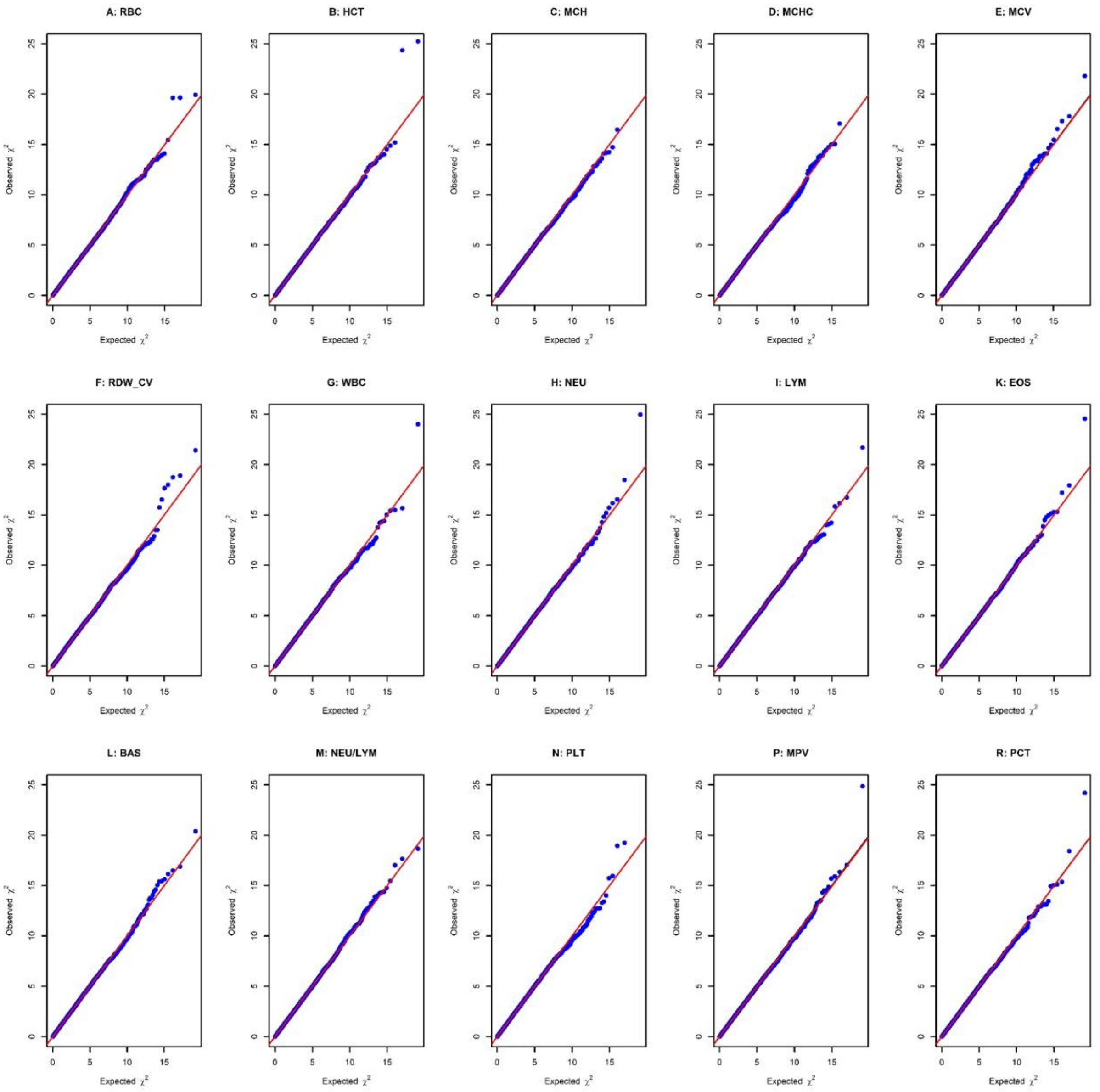

Supplementary Figure 1. Quantile-quantile (Q–Q) plots of genome-wide association studies (GWAS) after correcting p-values for the relevant traits. Red blood cell (A), Hematocrit (B), Mean corpuscular hemoglobin (C), Mean corpuscular hemoglobin concentration (D), MCV: Mean corpuscular volume (E), RBC volume distribution width coefficient of variation (F), White blood cell (G), Neutrophils (H), Lymphocytes (I), Eosinophils (K), Basophils (L), neutrophils/lymphocytes (M), Platelets (N), Mean platelets volume (P), Procalcitonin (R).
