# Supplementary material for: Estimates of genomic heritability and genome-wide association studies for blood parameters in Akkaraman sheep
Source: Sci Rep. 2022 Nov 2;12:18477. doi: 10.1038/s41598-022-22966-8 (PMC9630504; doi:10.1038/s41598-022-22966-8)
Supplement: Supplementary file 2 — Supplementary Information 2. [file 41598_2022_22966_MOESM2_ESM.pdf]

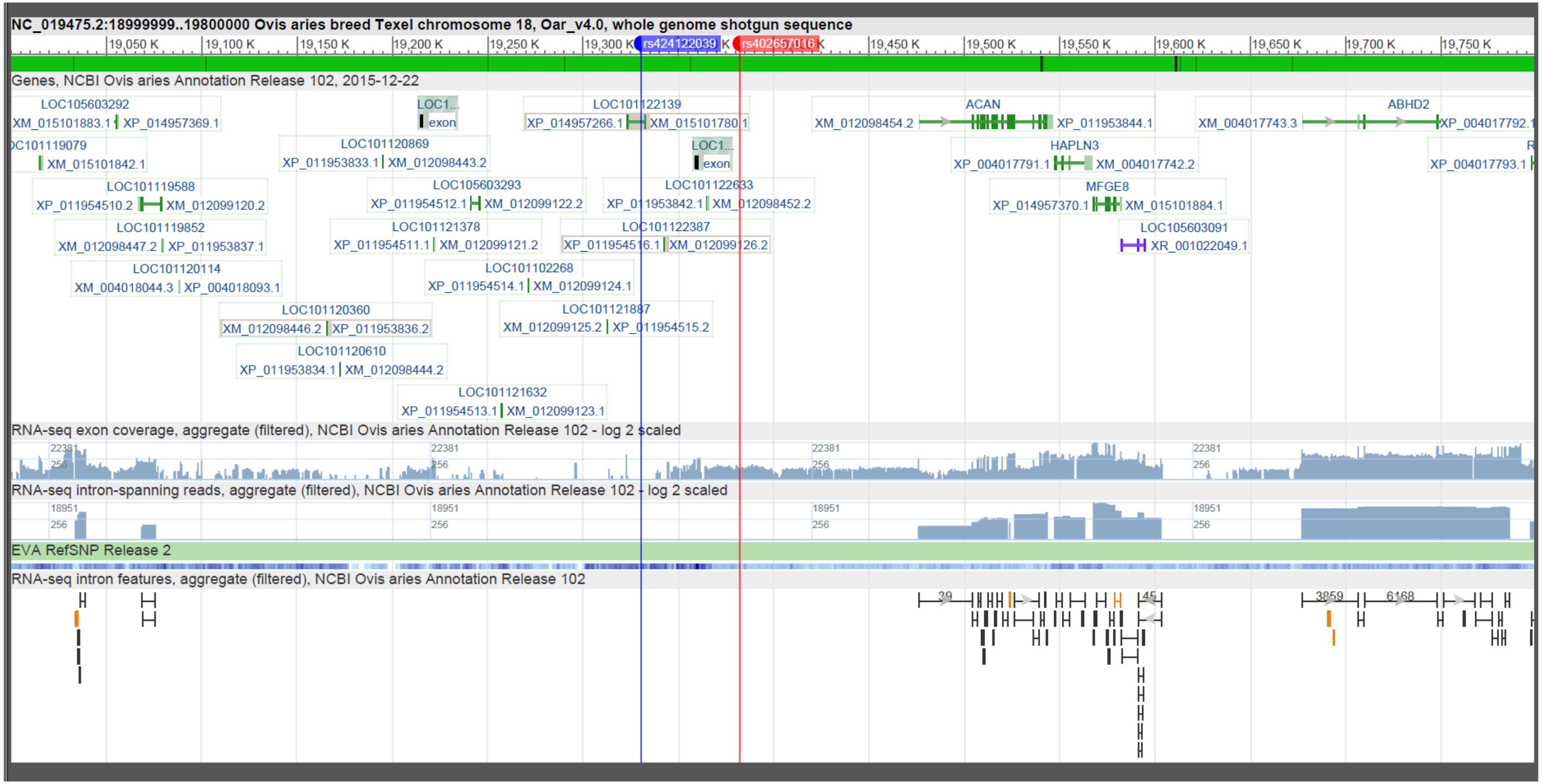

Supplementary Figure 2. ~800Kbp window on chromosome 18 including genes mainly populated by MYADM-like genes and significant SNPs.
