# Supplementary material for: Estimates of genomic heritability and genome-wide association studies for blood parameters in Akkaraman sheep
Source: Sci Rep. 2022 Nov 2;12:18477. doi: 10.1038/s41598-022-22966-8 (PMC9630504; doi:10.1038/s41598-022-22966-8)
Supplement: Supplementary file 4 — Supplementary Information 4. [file 41598_2022_22966_MOESM4_ESM.pdf]

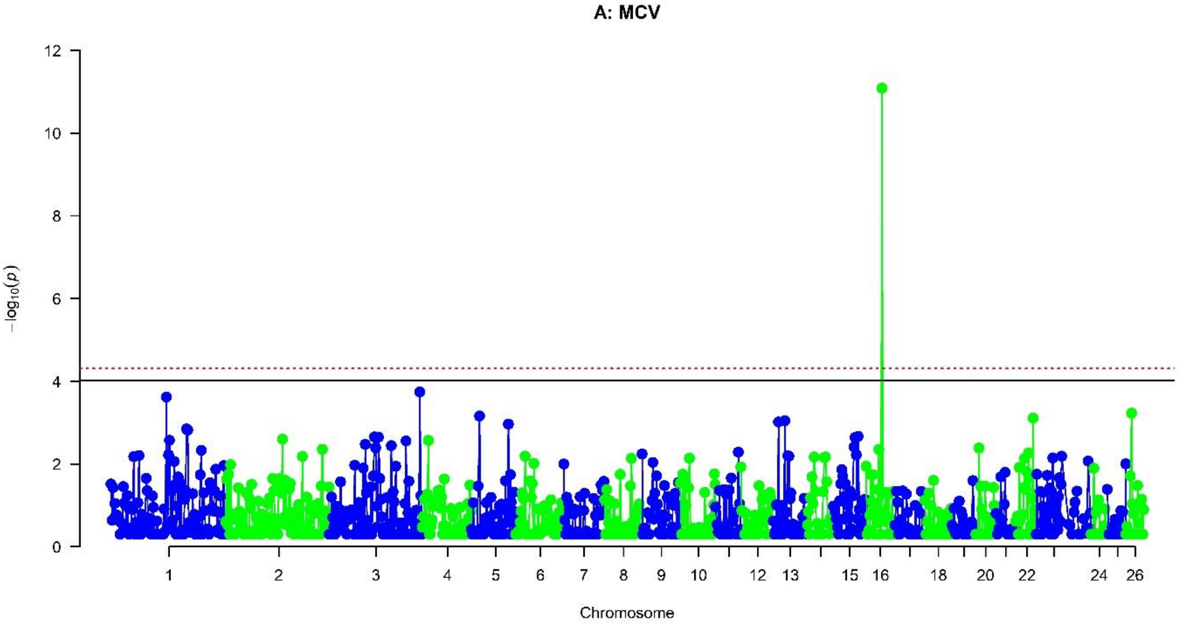

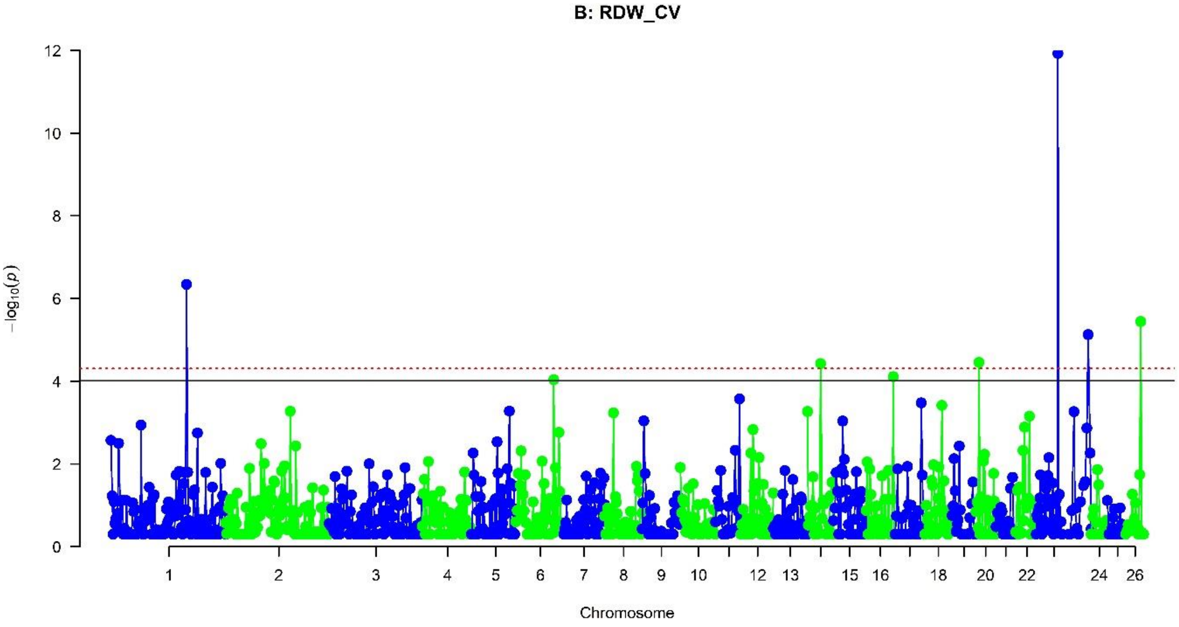

Supplementary Figure 4. Manhattan plots for associations between regions and mean corpuscular volume (MCV) and RBC volume distribution width coefficient of variation (RDW_CV). Red dashed horizontal lines indicate suggestive (0.10) significance and black horizontal line indicate genome-wide (0.05) significance with 1,038 windows.
