# Supplementary material for: Estimates of genomic heritability and genome-wide association studies for blood parameters in Akkaraman sheep
Source: Sci Rep. 2022 Nov 2;12:18477. doi: 10.1038/s41598-022-22966-8 (PMC9630504; doi:10.1038/s41598-022-22966-8)
Supplement: Supplementary file 5 — Supplementary Information 5. [file 41598_2022_22966_MOESM5_ESM.docx]

**Supplementary Table 1**. Summary statistics of the regional heritability analysis using 5 Mb-SNP windows.

| Traits | Chr. | Windows | | No. SNPs | Regional heritability (h^2^_reg_)  ± SE | LRT | -log_10_P | Significance |
| --- | --- | --- | --- | --- | --- | --- | --- | --- |
|  |  | **Start (Mb)** | **End (Mb)** |  |  |  |  |  |
| RBC | 1 | 7344208 | 9307030 | 40 | 0.08 ± 0.03 | 14.02 | 4.04 | Suggestive |
| MCH | 18 | 17367201 | 19881168 | 40 | 0.08 ± 0.03 | 16.99 | 4.72 | %5 |
| MCH | 1 | 270665267 | 273024024 | 41 | 0.10 ± 0.04 | 15.14 | 4.30 | %5 |
| MCHC | 18 | 17367201 | 19881168 | 40 | 0.11 ± 0.04 | 21.76 | 5.81 | %5 |
| MCHC | 23 | 34048698 | 35941216 | 40 | 0.08 ± 0.03 | 18.50 | 5.07 | %5 |
| MCHC | 1 | 186648768 | 189579918 | 41 | 0.11 ± 0.04 | 17.56 | 4.85 | %5 |
| MCHC | 1 | 270665267 | 273024024 | 41 | 0.09 ± 0.04 | 14.46 | 4.14 | Suggestive |
| MCV | 16 | 38337897 | 40409152 | 41 | 0.22 ± 0.05 | 45.36 | 11.08 | %5 |
| RDW_CV | 23 | 52374331 | 53898104 | 41 | 0.11 ± 0.03 | 49.15 | 11.92 | %5 |
| RDW_CV | 1 | 181528952 | 184214381 | 41 | 0.12 ± 0.04 | 24.12 | 6.34 | %5 |
| RDW_CV | 26 | 36053084 | 38212035 | 42 | 0.12 ± 0.04 | 20.14 | 5.44 | %5 |
| RDW_CV | 23 | 124788741 | 129824803 | 40 | 0.08 ± 0.03 | 18.74 | 5.12 | %5 |
| RDW_CV | 20 | 8048314 | 10232838 | 40 | 0.10 ± 0.04 | 15.81 | 4.45 | %5 |
| RDW_CV | 14 | 31605947 | 34642569 | 41 | 0.11 ± 0.04 | 15.68 | 4.42 | %5 |
| RDW_CV | 16 | 65658092 | 67555302 | 40 | 0.12 ± 0.05 | 14.30 | 4.10 | Suggestive |
| RDW_CV | 6 | 91488318 | 93388338 | 40 | 0.09 ± 0.04 | 13.99 | 4.03 | Suggestive |
| WBC | 19 | 12815694 | 15253598 | 40 | 0.10 ± 0.03 | 14.86 | 4.23 | Suggestive |
| NEU | 19 | 25433040 | 28153652 | 41 | 0.07 ± 0.03 | 15.01 | 4.27 | Suggestive |
| NEU | 6 | 50907002 | 53876295 | 40 | 0.08 ± 0.03 | 14.55 | 4.16 | Suggestive |
| LYM | 6 | 50907002 | 53876295 | 40 | 0.09 ± 0.04 | 16.55 | 4.62 | %5 |
| LYM | 9 | 19998734 | 21956766 | 41 | 0.08 ± 0.03 | 14.37 | 4.12 | Suggestive |
| EOS | 1 | 70205807 | 72129951 | 41 | 0.09 ± 0.03 | 17.81 | 4.91 | %5 |
| NEU/LYM | 6 | 50947002 | 53876295 | 40 | 0.08 ± 0.04 | 16.98 | 4.72 | %5 |
| NEU/LYM | 9 | 19998734 | 21956766 | 41 | 0.07 ± 0.03 | 16.91 | 4.70 | %5 |
| NEU/LYM | 2 | 175270952 | 177365713 | 41 | 0.07 ± 0.03 | 16.33 | 4.57 | %5 |
| NEU/LYM | 12 | 76953397 | 77800869 | 9 | 0.05 ± 0.03 | 15.27 | 4.33 | %5 |
| PCT | 1 | 240642734 | 243411104 | 41 | 0.26 ± 0.07 | 24.31 | 6.38 | %5 |
| PCT | 1 | 158852780 | 161267302 | 40 | 0.24 ± 0.07 | 23.00 | 6.09 | %5 |
| PCT | 1 | 217051601 | 218973343 | 40 | 0.30 ± 0.08 | 19.95 | 5.40 | %5 |
| PCT | 18 | 15061865 | 17227291 | 41 | 0.26 ± 0.08 | 18.07 | 4.97 | %5 |

LRT, likelihood ratio test; −log P = negative logarithm of nominal (i.e., uncorrected) p-value. Values of −log p greater than 4.31 and 4.01 correspond to Bonferroni-corrected genome-wide significance and suggestive levels of p < 0.05 and p < 0.10, respectively.
